# Loading ceftriaxone, vancomycin, and *Bifidobacteria bifidum* TMC3115 to neonatal mice could differently and consequently affect intestinal microbiota and immunity in adulthood

**DOI:** 10.1038/s41598-018-35737-1

**Published:** 2019-03-01

**Authors:** RuYue Cheng, JiaWen Guo, FangFang Pu, ChaoMin Wan, Lei Shi, HuaWen Li, YuHong Yang, ChengYu Huang, Ming Li, Fang He

**Affiliations:** 1 Department of Nutrition, Food Hygiene and Toxicology, West China School of Public Health and Healthy Food Evaluation Research Center, Sichuan University, 610041 Chengdu, Sichuan PR China; 2 Department of Pediatrics of Western China Second Hospital of Sichuan University, Key Laboratory of Birth Defects and Related Diseases of Women and Children, 610041 Chengdu, Sichuan PR China; 3 Department of Clinical Nutrition, West China Hospital, Sichuan University, 610041 Chengdu, Sichuan PR China; 4 Hebei Inatural Biotech Co., Ltd, 050000 Shijiazhuang, Hebei PR China

## Abstract

Recent studies have demonstrated that antibiotics/or probiotics administration in early life play key roles on modulating intestinal microbiota and the alterations might cause long-lasting consequences both physiologically and immunologically. We investigated the effects of early life ceftriaxone, vancomycin and *Bifidobacterium bifidum* TMC3115 (TMC3115) treatment on intestinal microbiota and immunity both in neonates and adults even after termination of antibiotics exposure. We found that ceftriaxone and vancomycin, but not TMC3115, significantly altered the intestinal microbiota, serum total IgE level, and the morphology and function of the intestinal epithelium in the neonatal mice. In the adult stages, the diversity and composition of the intestinal microbiota were significantly different in the antibiotic-treated mice, and ceftriaxone-treated mice exhibited significantly higher serum total IgE and OVA-specific IgE levels. TMC3115 significantly mitigated the alteration of intestinal microbiota caused by ceftriaxone not vancomycin. Antibiotics and TMC3115 can differently modulate intestinal microbiota and SCFAs metabolism, affecting the development and function of the immunity and intestinal epithelium to different degrees in neonatal mice. Neonatal ceftriaxone-induced abnormal intestinal microbiota, immunity and epithelium could last to adulthood partly, which might be associated with the enhancement of host susceptibility to IgE-mediated allergies and related immune responses, TMC3115 may protect against the side effects of antibiotic treatment, at least partly.

## Introduction

Allergic diseases are becoming a serious global public health problem, especially in industrialized countries, and are also sharply increasing, even in developing countries such as China^1,2^. Many epidemiological investigations have suggested a strong correlation between the increased allergies over the past several decades and decreased stimuli from environmental microbes, especially intestinal microbiota, caused by dynamic changes in lifestyle and diet^3^. This hypothesis has been supported by accumulating scientific evidences from various experimental and clinical studies that the intestinal microbiota might play an essential role in maintaining homeostasis and shaping the immune system. Furthermore, dysbiosis of the intestinal microbiota might be strongly associated with the pathology of various allergic and autoimmune diseases^4,5^. Therefore, intestinal microbiota could be a possible therapeutic target for the management of allergic diseases.

Among one of the pathological responses of many allergic diseases is the enhancement of serum IgE-mediated responses to common environmental antigens^6,7^. Therefore, IgE is considered a serum hallmark for allergies and practically used as one of the parameters to diagnose and monitor allergic diseases^8^. Mice raised as germ-free from birth exhibited an enlarged, thin-walled, and fluid-filled cecum, altered kinetics of intestinal epithelial cells (IECs) turnover, and poorly developed lymphatic organs compared with conventionally raised mice^9,10^. This indicates that indigenous microbiota play a crucial inductive role in the intestinal tract and immune system development during early postnatal life. Furthermore, germ-free and antibiotic-treated mice showed higher serum IgE levels than conventional mice^11,12^, and the serum IgE levels started increasing in germ free mice early in life, around 3–4 weeks of age, correlating with the time of weaning^13^. These results suggest that the intestinal microbiota built in early life might be significantly involved in the development of allergic disorders^14^. However, additional studies are required to elucidate how intestinal microbiota in early life could alter host IgE-related immunity, even as adults.

Antibiotics have long been used to protect humans from various infectious diseases by killing harmful/or infective microbes, significantly contributing to human health. However, an overload of antibiotics, especially those with a broad spectrum, might substantially impair the quality and quantity of intestinal microbiota, inducing dysbiosis by altering the predominant microbe composition, causing antibiotic-resistant infective agents, and influencing intestinal microbe metabolism^15–17^. Recent epidemiological and animal studies have also demonstrated that antibiotic exposure in early life may cause dysfunctional IECs and local and systemic immunological disorders in the host^18–21^. In our previous study, ceftriaxone and vancomycin were found to cause apparent dysbiosis of the fecal microbiota in neonatal mice accompanied by adverse changes in the morphology of IECs and their proliferation and differentiation characterized, which performed as villi and crypts atrophy and decreased Ki67-/Muc2-positive cells^22^. In order to elucidate the underlying mechanisms of antibiotic damage to the intestinal microbiota, and how antibiotics influence the physiological and immunological homeostasis of the intestine, it remains necessary to characterize or profile the antibiotics that alter intestinal microbiota in more detail.

Probiotics are defined as “live microorganisms which when administered in adequate amounts confer a health benefit to the host”^23^ (FAO/WHO, 2002). Lactobacillus and bifidobacteria have been proven to be a substantial reservoir for microorganisms with probiotic attributes. Numerous *in vivo* and *vitro* studies have confirmed the beneficial activity of some exogenous species from lactobacilli and bifidobacteria in human health^24^. As probiotic agents, bifidobacteria have been well documented for their efficacy in the prevention and alleviation of various diseases or disorders including gastrointestinal and allergic diseases^25^. Oral intake of bifidobacteria may promote epithelial barrier function, and reduced bacterial translocation in mice with ischemia and reperfusion injury, even preventing dextran sulfate sodium -induced colitis^26–28^. Several studies have also indicated that some selective probiotic strains greatly promote the growth of neonatal mice through enhancing the morphological development of IECs and functional differentiation, at least partly^29,30^. However, it remains unclear whether probiotics, especially bifidobacteria, which usually colonize in the intestine of host animals from early life, can alleviate the side effects of antibiotics on the host animal in the neonate and adult stages.

As possible underlying mechanisms, accumulated evidence indicates that some species of bifidobacteria suppress the immune responses of type 2T helper (Th2) cells *in vitro* and in animal studies by promoting the production of IL-10 and IL-12^31^, improving the development of regulatory T (Treg) cells^32^, and reducing serum IgE and IL-4 levels^33^. Our previous study demonstrated that the selection of *Bifidobacterium bifidum* TMC3115 (TMC3115) as a new selective probiotic inhibited serum IgE production caused by ovalbumin (OVA) and suppressed the expression of splenic IL-6 and IL-12, and tumor necrosis factor (TNF)-α mRNA^34^.Therefore, we wanted to determine if TMC3115 could also protect adult hosts from allergic disorders caused by antibiotic-altered intestinal microbiota and immune function in their early life.

This study aimed to demonstrate whether antibiotic-altered intestinal microbiota in early life could consequently affect the susceptibility to IgE-mediated allergic disorders in adults, and explore the protective effects of bifidobacteria. In the present study, BALB/c mice were administered ceftriaxone, vancomycin, and TMC3115 for 3 weeks, from birth to weaning. After the intervention, the mice were sensitized intraperitoneally with the food antigen OVA. Mice were sacrificed at postnatal day 21 (weaning) and day 56 (adult), respectively and their body weight, spleen index, fecal microbiota, ileal villi depth, colonic crypt depth, intestinal Ki67- and Muc2-positive cells, serum total IgE level, OVA-specific IgE level, TNF-α, IL-2, IL-4, IL-6, IL-10, IL-12, IL-17A and IFN-γ levels, and splenic CD4+ T cell subsets were examined to determine changes in gut microbiota, intestinal morphology and cell function, and the immune response to OVA.

## Results

### Growth rate and spleen index of mice

**Figure 1:**
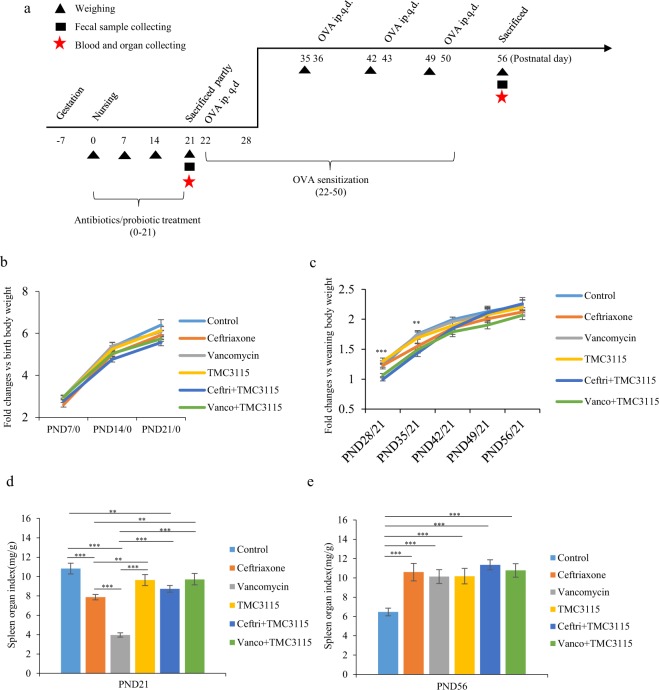
Body weight, spleen index changes of mice. (**a**) Experimental design. There were 18 mice per group when the experiment began, and 6 mice per group were sacrificed to collect blood, spleen and intestine when at PND21, thus there were 12 mice per group immunized by OVA and sacrificed in the end of this study. ip., intraperitoneal, q.d., one time per day. (**b**,**c**) Fold changes of murine body weight at PND7, 14, and 21 verse birth were calculated and taken as the growth rate before weaning. Fold changes of murine body weight at PND28, 35, 42, 49 and 56 verse PND21 were calculated and taken as the growth rate after weaning (n = 6–12/group). (**d**,**e**) To evaluate the development of immune organ after neonatal antibiotics or probiotic treatment, the spleen index of mice at PND 21 was calculated. Organ index of spleen is displayed by the ratio of the respective mass of spleen to the body weight at PND21/PND56 (n = 6/group). mg: milligram, g: gram. The two ends of the horizontal line represent the two compared groups, the asterisk on the line represents significant difference between groups, *p < 0.05, **p < 0.01, ***p < 0.001, for intergroup comparisons. TMC3115, *Bifidobacterium bifidum* TMC3115; Ceftri, Ceftriaxone; Vanco, Vancomycin. PND, postnatal day.

At postnatal day (PND) 21, the body weights of the mice in all groups were steadily increasing, and no clinical side effects or mortality was observed. There was no significant difference in the growth rate of mice among groups (Fig. 1b). The spleen index of mice at PND 21 was calculated (Fig. 1d), and that of the mice exposed to ceftriaxone and vancomycin was significantly decreased compared with the control *(P* < 0.001). Furthermore, vancomycin reduced the spleen index considerably more than ceftriaxone *(P* < 0.001). No significant body weight or spleen index differences were found in the TMC3115 group or the vancomycin (vanco) + TMC3115 group compared with the control group. However, the spleen index of the mice treated with ceftriaxone (ceftri) + TMC3115 was decreased significantly compared with the control group *(P* < 0.01).

After weaning and discontinued antibiotic or TMC3115 treatment, body weight was measured at PND 28, 35, 42, 49 and 56. However, the growth rate of mice in the ceftri + TMC3115 and vanco + TMC3115 groups at PND 28 and 35 showed a significant reduction compared with the other groups (Supplementary Table S1). Nevertheless, owing to the body weights of mice gradually increasing with age, no significant difference was observed among all the groups when comparing the fold changes of body weights at PND 42, 49, and 56 versus weaning (Fig. 1c). Similarly, all spleens of the sacrificed mice were weighed, and the organ index was calculated. The spleen index of mice in the control group was significantly lower than in the other groups (all *P* < 0.001), whereas there was no significant difference between mice fed with ceftriaxone, vancomycin, TMC3115, ceftri + TMC3115, and vanco + TMC3115 (Fig. 1e).

### Histological analysis of intestinal morphology

**Figure 2:**
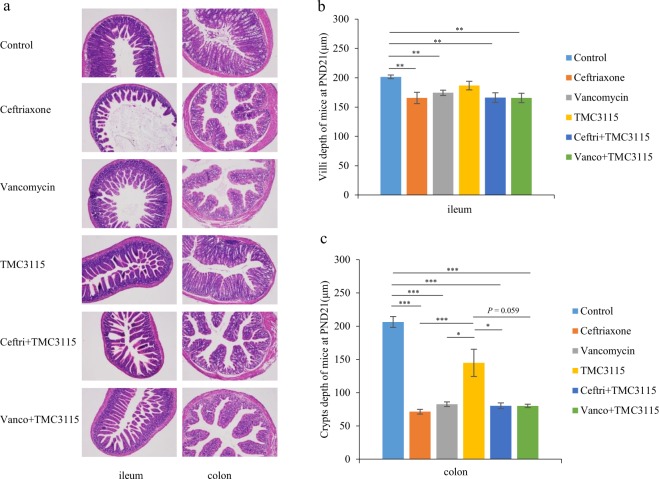
Intestinal tissue injury of mice at PND21. (**a**) To evaluate the potential intestinal tissue injury caused by antibiotics or probiotic, the staining profiles by H&E of ileum and colon in mice of six groups were displayed (n = 6/group). Scale bars: 100 μm. (**b**) The depth of murine ileal villi at PND21 (n = 6/group). (**c**) The depth of murine colonic crypts at PND21 (n = 6/group). The two ends of the horizontal line represent the two compared groups, the asterisk on the line represents significant difference between groups, *p < 0.05, **p < 0.01, ***p < 0.001, for intergroup comparisons.TMC3115*, Bifidobacterium bifidum* TMC3115; Ceftri, Ceftriaxone; Vanco, Vancomycin. PND, postnatal day.

Histological cross-sections of the ileum and colon from mice at PND 21 are shown in Fig. 2a. The ileum and colon in the control mice and TMC3115–treated mice were well-shaped with elongated villi and crypts lined with columnar epithelia. However, those of the antibiotic-treated mice lost regular villi structure with a lack of regularly distributed columnar epithelial cell nuclei lining the villi and shortened and irregular villi and crypts. The ileal villi lesions and colonic crypt atrophy in mice treated with ceftriaxone, vancomycin, ceftri + TMC3115, and vanco + TMC3115 were significantly less compared with the control group. The colonic crypts in mice treated with TMC3115 were significantly deeper than those treated with ceftriaxone, vancomycin, and ceftri + TMC3115, and a similar tendency toward deeper colonic crypts was seen in the vanco + TMC3115 group. There was no significant difference in the depth of villi or crypts between the control and TMC3115 group (Fig. 1b,c). At PND 56, no significant differences among the tested mice in the depth of their ileal villi and colonic crypts were observed (Supplementary Fig. S1a,b).

### Development and differentiation of IECs

**Figure 3:**
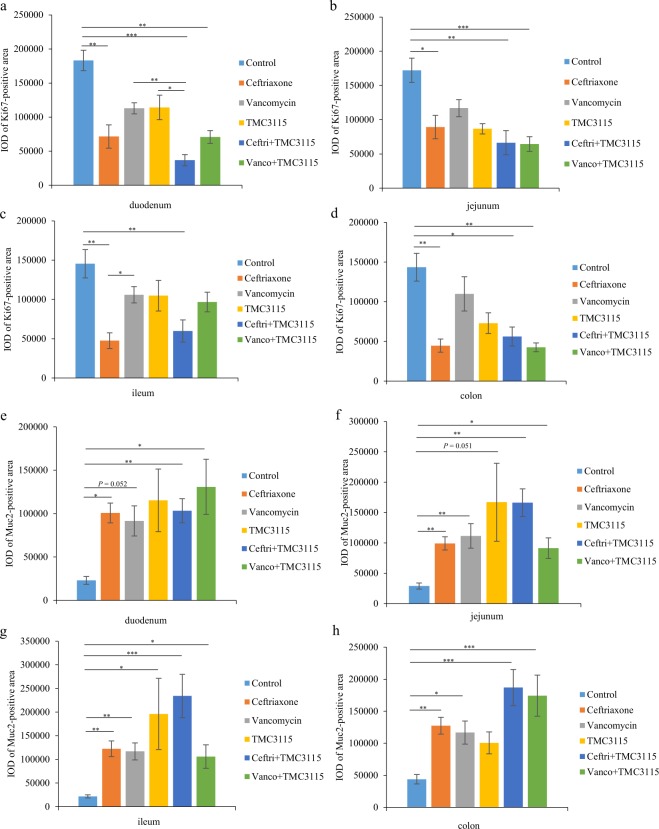
Immunohistochemical analysis of intestinal epithelial cells (IECs) impairments. (**a**–**d**) To examine the effect of antibiotics or probiotic treatment on the proliferative activity of IECs in neonatal mice, the Ki67-positive cells of mice at PND 21 were investigated. Ki67-positive area of the four intestinal segments (duodenum, jejunum, ileum, and colon) in mice at PND21 (n = 6/group). (**e**–**h**) To determine whether the production of mucin proteins secreted by IECs were influenced by antibiotics or probiotic treatment, the Muc2-positive cells were measured. Muc2-positive area of the four intestinal segments (duodenum, jejunum, ileum, and colon) in mice at PND21. IOD: integral optical density (n = 6/group). The two ends of the horizontal line represent the two compared groups, the asterisk on the line represents significant difference between groups, *p < 0.05, **p < 0.01, ***p < 0.001, for intergroup comparisons. TMC3115*, Bifidobacterium bifidum* TMC3115; Ceftri, Ceftriaxone; Vanco, Vancomycin. PND, postnatal day.

At PND 21, in all four intestine segments (duodenum, jejunum, ileum, and colon), the proliferative activity of IECs was markedly attenuated in mice treated with ceftriaxone and ceftri + TMC3115 according to the reduction of Ki67-positive cells compared with the control group. In the duodenum, jejunum, and colon, significantly decreased Ki67-positive cells were observed in the vanco + TMC3115 group compared with control group. Furthermore, in the duodenum, the reduction of Ki67-positive cells in the ceftri + TMC3115 group was more significant than in the TMC3115 and vancomycin group. In the ileum, the number of Ki67-positive cells in mice treated with ceftriaxone was lower than those treated with vancomycin (Fig. 3a–d). The production of Muc2 in all four intestine segments (duodenum, jejunum, ileum, and colon) was significantly higher in mice treated with ceftriaxone, ceftri + TMC3115, and vanco + TMC3115 than the control. Additionally, in the jejunum, ileum, and colon, significantly decreased Muc2-positive cells mice treated with vancomycin showed a significant increase of compared with control group. The Muc2-positive cells in the duodenum of mice treated with vancomycin compared with the control tended to be increased, and a similar tendency was observed in the comparison of Muc2-positive cells in the jejunum between TMC3115 and the control group. However, Muc2 production in the ileum was significantly increased compared with the control group (Fig. 3e–h). At PND 56, however, no significant differences in the amount of Ki67- or Muc2-positive cells were observed among groups (Supplementary Fig. S1c,d).

### Total fecal bacterial count by quantitative polymerase chain reaction (qPCR)

**Figure 4:**
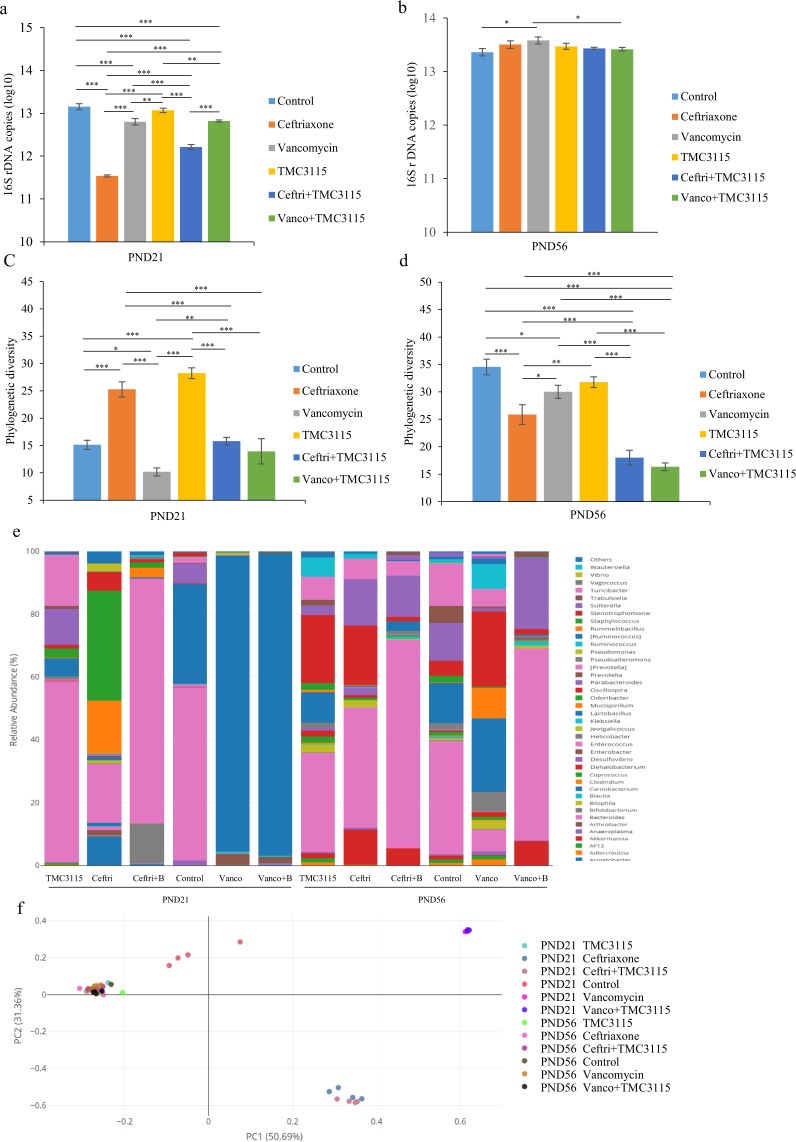
Microbial community composition alterations of mice at PND21 and PND56. (**a**,**b**) Total extracted DNA of bacteria from feces was amplified by quantitative PCR and the 16S rDNA copies were calculated as the fecal bacterial numbers. The log10 value of the original data are shown (n = 8/group). (**c**,**d**) Alpha-diversity with phylogenetic diversity of intestinal microbiota is shown (n = 4/group). (**e**) Genus level phylogenetic classification of 16S rRNA gene frequencies in feces collected from naïve control and neonatally antibiotic/probiotic-treated animals (n = 4/group). Each bar represents one group. Rare taxa (<1%) are classified into “others”. (**f**) Bacterial communities from feces of naive control and neonatally antibiotic/probiotic-treated mice were compared using principal coordinate analysis (PCoA) (n = 4/group).The PCoA analysis is based on the weighted Unifrac distance. The two ends of the horizontal line represent the two compared groups, the asterisk on the line represents significant difference between groups, *p < 0.05, **p < 0.01, ***p < 0.001, for intergroup comparisons. TMC3115*, Bifidobacterium bifidum* TMC3115; Ceftri, Ceftriaxone; Vanco, Vancomycin. PND, postnatal day.

At PND 21, ceftriaxone significantly reduced fecal bacterial numbers compared with the control, vancomycin, TMC3115, ceftri + TMC3115 and vanco + TMC3115 group (all *P* < 0.001). Vancomycin significantly reduced fecal bacterial numbers compared with control, TMC3115, ceftri + TMC3115 group (*P* < 0.001, *P* < 0.01 and *P* < 0.001, respectively). No significant difference between the vancomycin and vanco + TMC3115 group were observed. The fecal bacterial numbers in mice treated with TMC3115 was significantly higher than that of ceftri + TMC3115 and vanco + TMC3115-treated mice (*P* < 0.01 and *P* < 0.001, respectively). However, TMC3115 did not significantly affect the fecal bacterial numbers compared with the control. The fecal bacteria were significantly higher in the mice treated with vanco + TMC3115 than those exposed to ceftri + TMC3115 (*P* < 0.001) (Fig. 4a).

At PND 56, the fecal bacterial numbers of the mice treated with vancomycin was significantly increased compared with the control and vanco + TMC3115 group (both *P* < 0.05) (Fig. 4b).

### Fecal microbiota analyzed with next-generation sequencing (NGS)

At PND 21, there was no significant difference in the phylogenetic diversity of the fecal microbiota among the control, ceftri + TMC3115, and vanco + TMC3115 groups. However, the phylogenetic diversity of the fecal microbiota was significantly lower in those treated with vancomycin compared with the control (*P* < 0.05). On the other hand, ceftriaxone and TMC3115 significantly increased the phylogenetic diversity of fecal microbiota (both *P* < 0.001) (Fig. 4c).

At PND 56, ceftri + TMC3115 and vanco + TMC3115 groups exhibited a reduced phylogenetic diversity that was significantly lower than the other four groups (*P* < 0.001). However, the phylogenetic diversities in the control and TMC3115 groups were higher than the ceftriaxone group (*P* < 0.001 and *P* < 0.01, respectively) (Fig. 4d).

At PND 21, *Actinobacteria* and *Firmicutes* significantly increased in the feces of mice treated with ceftriaxone and ceftri + TMC3115 compared with the control *(P* < 0.01); *Proteobacteria* significantly increased in the feces of the mice treated with ceftriaxone, vancomycin, and vanco + TMC3115 group *(P* < 0.05*, P* < 0.01 and *P* < 0.01, respectively); and *Bacteroidetes* significantly decreased in both mice treated with and without TMC3115. Conversely, *Bacteroidetes* significantly increased and *Firmicutes* significantly decreased in mice treated with TMC3115 *(P* < 0.05 and *P* < 0.01, respectively). No significant differences between *Firmicutes* and *Actinobacteria* in comparisons of vancomycin vs. control or vanco + TMC3115 vs. control was observed (Table 1).

**Table 1:** Mean relative abundance of different taxa at phylum level.

Stage	Phylum	Control	Ceftriaxone	Vancomycin	TMC3115	Ceftri + TMC3115	Vanco + TMC3115
PND21	*Actinobacteria*	0.02%	1.72%**	<0.01%	0.08%	12.36%**	0.14%
	*Bacteroidetes*	65.71%	5.38%***	0.15%**	78.26%*	1.08%***	0.11%**
	*Firmicutes*	27.39%	71.01%**	29.2%	12.34%**	83.39%**	28.77%
	*Proteobacteria*	5.60%	21.17%*	70.64%***	8.92%	3.00%	70.98%***
PND56	*Actinobacteria*	0.18%	0.05%	0.36%	0.24%	<0.01%	<0.01%
	*Bacteroidetes*	79.33%	43.30%***	58.55%***	38.33%***	63.96%***	57.33%***
	*Deferribacteres*	0.04%	0.01%	1.62%*	0.21%	<0.01%	0.04%
	*Firmicutes*	15.52%	48.29%**	37.41%**	57.20%**	25.58%*	28.64%**
	*Proteobacteria*	3.53%	4.18%	1.72%	2.47%	6.23%	9.16%**
	*Verrucomicrobia*	0.80%	4.03%	0.01%	0.35%	3.57%*	4.77%*

n = 4/group; *p < 0.05, **p < 0.01, ***p < 0.001, comparing with control. TMC3115*, Bifidobacterium bifidum* TMC3115; Ceftri, Ceftriaxone; Vanco, Vancomycin; PND, postnatal day.

At PND 56, compared with the control, *Bacteroidetes* significantly decreased and *Firmicutes* significantly increased in all groups *(P* < 0.001); *Deferribacteres* significantly increased in the mice treated with vancomycin (*P* < 0.001); *Proteobacteria* significantly increased in the vanco + TMC3115 group; and *Verrucomicrobia* significantly increased in the ceftri + TMC3115 and vanco + TMC3115 groups. No significant difference in *Actinobacteria* between the control and other groups (Table 1).

At PND 21, *Bacteroides*, *Lactobacillus*, and *Parabacteroides* were the three predominant fecal bacteria in the control. The relative abundance of *Lactobacillus* in the TMC3115 group significantly decreased, and the predominant bacteria were found to be *Bacteroides*, *Prevotella*, and *Parabacteroides*. Differences in *Bacteroides* and *Parabacteroides* between the control and TMC3115 group were not significant. However, *Bacteroides* in ceftriaxone, vancomycin, ceftri + TMC3115, and vanco + TMC3115 groups significantly decreased compared with the control. *Enterococcus*, *Staphylococcus*, and *Rummeliibacillus* were mainly found in the ceftriaxone group and became the predominant fecal bacteria. *Enterococcus* and *Bifidobacterium* in the ceftri + TMC3115 group significantly increased to become the predominant fecal bacteria. For both the vancomycin and vanco + TMC3115 group, *Lactobacillus* were significantly enriched and became the most predominant bacteria (Fig. 4e, Table 2, Supplementary Table S2).

**Table 2:** Mean relative abundance of different taxa at genus level.

Stage	Genus	Control	Ceftriaxone	Vancomycin	TMC3115	Ceftri + TMC3115	Vanco + TMC3115
PND21	*Acinetobacter*	<0.01%	9.45%*	0.02%	0.06%	0.44%	0.2%
	*Arthrobacter*	<0.01%	1.64%**	<0.01%	<0.01%	0.08%	<0.01%
	*Bacteroides*	55.29%	1.21%**	0.14%**	57.78%	0.12%*	0.08%**
	*Bifidobacterium*	<0.01%	<0.01%	0.03%	<0.01%	12.74%**	0.42%
	*Enterococcus*	0.95%	18.91%*	0.13%	0.3%	78.04%***	0.02%
	*Lactobacillus*	31.8%	1.22%*	94.36%***	5.86%*	0.12%*	96.29%***
	*Odoribacter*	<0.01%	<0.01%	<0.01%	2.85%**	<0.01%	<0.01%
	*Oscillospira*	0.11%	<0.01%	<0.01%	1.21%*	0.01%	0.01%
	*Parabacteroides*	6.46%	0.24%**	0.03%**	11.32%	<0.01%**	0.01%**
	*Prevotella*	0.11%	<0.01%	<0.01%	1.13%***	<0.01%	<0.01%
	*Rummeliibacillus*	0.01%	16.55%**	<0.01%	<0.01%	3.14%	<0.01%
	*Staphylococcus*	0.06%	35.14%***	<0.01%	0.07%	1.68%**	0.06%
	*Stenotrophomonas*	1.28%	5.91%*	<0.01%	<0.01%	1.01%	0.18%
PND56	*AF12*	1.06%	<0.01%*	1.21%	1.25%	<0.01%	<0.01%*
	*Akkermansia*	1.34%	11.33%*	0.09%	1.59%	5.46%**	7.78%**
	*Anaeroplasma*	0.29%	0.51%	1.34%*	0.32%	<0.01%	0.09%
	*Bacteroides*	36.3%	38.41%	6.97%*	31.73%	66.4%*	61.28%*
	*Bifidobacterium*	<0.01%	<0.01%	<0.01%	0.01%	<0.01%	<0.01%
	*Bilophila*	0.56%	2.26%**	2.87%**	2.61%***	0.37%	0.82%
	*Blautia*	0.49%	<0.01%	<0.01%	0.26%	0.65%	1.55%*
	*Dehalobacterium*	0.37%	0.79%	1.67%**	1.88%**	0.01%	<0.01%
	*Enterococcus*	<0.01%	0.04%	<0.01%	0.02%	0.01%	<0.01%
	*Helicobacter*	2.06%	0.04%**	6.36%***	1.73%	1.13%	0.59%**
	*Lactobacillus*	12.86%	0.51%	23.27%	9.55%	3.2%	0.39%
	*Mucispirillum*	<0.01%	0.03%	9.86%**	0.85%	<0.01%	0.05%
	*Odoribacter*	1.95%	<0.01%*	0.33%*	2.05%	<0.01%*	<0.01%*
	*Oscillospira*	4.91%	18.98%	23.91%**	21.67%*	1.36%	1.7%
	*Parabacteroides*	11.97%	14.71%	0.94%*	3.09%	13.2%	22.65%*
	*Prevotella*	5.38%	0.01%*	0.54%	1.8%	<0.01%*	<0.01%*
	*Ruminococcus*	1.33%	1.55%	7.93%**	6.14%	<0.01%	0.13%
	*Sutterella*	1.4%	<0.01%***	0.71%	0.05%**	1.6%	<0.01%**
	*Trabulsiella*	<0.01%	<0.01%	0.9%	<0.01%	1.02%***	1.8%***

n = 4/group; *p < 0.05, **p < 0.01, ***p < 0.001, comparing with control. TMC3115*, Bifidobacterium bifidum* TMC3115; Ceftri, Ceftriaxone; Vanco, Vancomycin; PND, postnatal day.

At PND 56, *Bacteroides*, *Lactobacillus*, and *Parabacteroides* were still the predominant bacteria in the control. *Oscillospira* significantly increased and became the predominant bacteria in the TMC3115 group together with *Bacteroides* and *Parabacteroides*. *Bacteroides, Oscillospira*, and *Parabacteroides* were the predominant bacteria in the ceftriaxone group with no significant differences compared with the control. *Akkermansia* were found more in the ceftriaxone group. *Oscillospira* and *Mucispirillum* significantly increased and became the predominant bacteria in the vancomycin group. There was no significant difference in *Lactobacillus* between the control and vancomycin groups. *Bacteroides* in the vancomycin group significantly decreased. *Bacteroides*, *Parabacteroides*, and *Akkermansia* significantly increased and became the predominant bacteria in the ceftri + TMC3115 and vanco + TMC3115 groups (Fig. 4e, Table 2, Supplementary Table S3).

Analysis of the microbial beta-diversity with PCoA was based on weighted Unifrac distance to clarify the differences in relative bacterial abundance and evolution between groups. In fact, 50.69% of the variability was explained on axis PC1, emphasizing the substantial shift in community composition that resulted from treatment with antibiotics alone, or antibiotics together with TMC3115. It was clear that early life antibiotic treatment displayed the maximum effect on community composition, irrespective of TMC3115 supplementation. On the other hand, PCoA analysis also showed that all of the tested adult mice clustered into one area, irrelative to OVA sensitization. (Fig. 4f).

### Modifications of cecal SCFA levels

At PND21, cecal acetate was detected in all of the tested mice, and isobutyrate, propionate, butyrate, isovalerate, valerate, and caproate were detected only in control and TMC3115 groups. The cecal acetate levels in control and TMC3115 groups were significantly higher than other groups. Moreover, cecal acetate, isobutyrate, butyrate, isovalerate, valerate, and caproate in the neonatal mice in the TMC3115 group were significantly higher than those in the control group. However, there was no significant difference in propionate levels in the neonatal mice between the two groups (Table 3). At PND56, no significant differences were observed for all seven types of SCFAs in the adult mice among all the groups (Supplementary Table S4).

**Table 3:** Short chain fatty acids in cecal contents of mice at PND21.

SCFAs (μg/g)	Control	Ceftriaxone	Vancomycin	TMC3115	Ceftri + TMC3115	Vanco + TMC3115
Acetic acid	572.41 ± 132.20^###^	329.72 ± 30.94**^, ###^	342.3 ± 56.69**^, ###^	1030.23 ± 148.42**	398.3 ± 66.90**^, ###^	304.43 ± 49.43***^, ###^
Propionic acid	186.60 ± 34.47	ND	ND	266.87 ± 122.26	ND	ND
Isobutyric acid	19.45 ± 8.23	ND	ND	44.21 ± 7.06**	ND	ND
Butyric acid	191.86 ± 123.23	ND	ND	1279.81 ± 343.33**	ND	ND
Isovaleric acid	9.51 ± 1.09	ND	ND	26.76 ± 2.63***	ND	ND
Valeric acid	0.21^a^	ND	ND	60.93 ± 4.45**	ND	ND
Caproic acid	0.20^a^	ND	ND	1.41 ± 0.16**	ND	ND

n = 4, per group. Data are expressed as mean ± standard deviation (SD) of mean. The letter “a” represents that there’s a missing SD in valeric acid and caproic acid of control group because only one sample had been detected. **P < 0.01, ***P < 0.001, compared with control group; ^###^*P* < 0.001, compared with TMC3115 group. SCFAs: short chain fatty acids; TMC3115*, Bifidobacterium bifidum* TMC3115; Ceftri, Ceftriaxone; Vanco, Vancomycin;PND, postnatal day, μg: microgram; ND, not detected.

### Total serum IgE, OVA-specific IgE and cytokines

**Figure 5:**
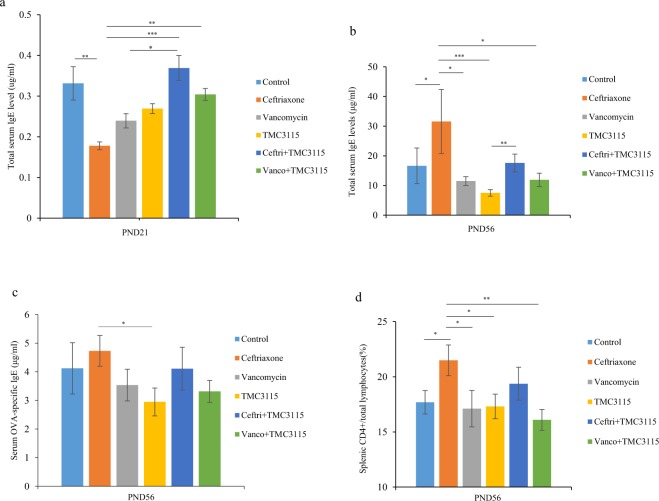
Immune response of mice at different stages. (**a**) Serum total IgE levels of mice at PND 21 (n = 6/group). μg: microgram, ml: milliliter. (**b**) Serum total IgE levels of mice at PND56 (n = 12/group). (**c**) Serum OVA-specific IgE of mice at PND56 (n = 12/group). (**d**) The percentage of the CD4+ T cells/lymphocytes in spleen are shown (n = 12/group). pg: picogram. The two ends of the horizontal line represent the two compared groups, the asterisk on the line represents significant difference between groups, *p < 0.05, **p < 0.01, ***p < 0.001, for intergroup comparisons. TMC3115*, Bifidobacterium bifidum* TMC3115; Ceftri, Ceftriaxone; Vanco, Vancomycin. PND, postnatal day.

At PND 21, loading ceftriaxone on neonatal mice for three weeks induced significantly lower level of total serum IgE than those in the control, ceftri + TMC3115 and vanco + TMC3115 groups respectively (*P* < 0.01, *P* < 0.001 and *P* < 0.01, respectively). The total serum IgE level of the tested mice in vancomycin group was significantly lower than those of the mice in ceftri + TMC3115 group (*P* < 0.05, Fig. 5a). At PND 56, neonatal ceftriaxone treatment induced the highest level of total serum IgE, being higher than that of the control, vancomycin, TMC3115, vanco + TMC3115 groups respectively (*P* < 0.05, *P* < 0.05, *P* < 0.001 and *P* < 0.05, respectively). No significant difference was observed between the ceftriaxone and ceftri + TMC3115 group. Moreover, total serum IgE was significantly decreased in the mice treated with TMC3115 compared with ceftri + TMC3115 *(P* < 0.01). The total serum IgE was less in the mice treated with TMC3115 in early life compared with those of the control group (Fig. 5b).

Mice treated with ceftriaxone in early life showed significant higher serum OVA-specific IgE level than those exposed to TMC3115 (*P* < 0.05). However, no significant difference among other groups although the serum OVA-specific IgE in ceftriaxone group was the highest (Fig. 5c).

No significant differences in serum cytokines among groups, including TNF-α, IL-2, IL-4, IL-6, IL-10, IL-12, IL-17A and IFN-γ (Supplementary Fig. S1e).

### Differentiation of the splenic CD4+ T cells subsets

At PND 56, the percentage of splenic CD4+ T cells was higher after the administration of ceftriaxone compared with control, vancomycin, TMC3115, and vanco + TMC3115 (*P* < 0.05, *P* < 0.05, *P* < 0.05 and *P* < 0.01, respectively) (Fig. 5d).

## Discussion

*Bifidobacterium* are among the first microbes to colonize the human gastrointestinal tract and are believed to exert positive health benefits on their host. *Bifidobacteria* naturally occur in a range of ecological niches that are either directly or indirectly connected to the animal gastrointestinal tract, such as the human oral cavity, the insect gut, and sewage^35^. Several recent studies have investigated the impact on the gut microbiome by bifidobacterial cross-feeding of carbohydrates. These studies have demonstrated that some bifidobacterial communities, especially *B. longum* and *B. bifidum*, can degrade large complex polysaccharides, including plant-derived polysaccharides and host-derived carbohydrates such as mucin, into more simple sugars which are in turn then available to other members of the gut microbiota^36^.

*Bifidobacterium bifidum* TMC3115 (TMC3115) was originally isolated from healthy infants and can adhere well to human IECs and mucosa in a strain-dependent manner without inflammation^37^. TMC3115 exhibited much stronger inhibitory effects against IgE-mediated degranulation of RBL-2H3 cells *in vitro* than those of other bifidobacteria, especially those taxonomically identified as *B. adolescentis*^38^ and can significantly suppress the increase of serum IgE caused by OVA immunization in mice^34^. Whole genome information regarding TMC3115 (https://www.ncbi.nlm.nih.gov/nuccore/1249614599) indicates that it has encoding loci for the structure of cell surface pili and the utilization of mucin and human milk oligosaccharide (HMO), as does *B.bifidum* PRL2010, a well-known probiotic strain with widely documented health-promoting effects^39^. Furthermore, whole genome information of this bacterium indicates that it has encoding loci for resistance to β-lactam antibiotics. However, this bacterium is sensitive to vancomycin *in vitro* (Data not publication). These previous studies hypothesized that TMC3115 could protect host animals from the side effects of some antibiotics in the neonate and adult stages by cross-feeding with other intestinal microbes, thus affecting host immunity.

In the present study, ceftriaxone, vancomycin, and the probiotic strain TMC3115 were administered alone or combined with each other after birth to weaning. Such early life exposure to antibiotics and probiotics did not cause any apparent clinical symptoms as were observed in the previous study^22^. However, the apparent damages to intestinal morphology including ileal villi, colonic crypts depth, and the maturation of IECs were as found in the previous study^22^. Compared with vancomycin, ceftriaxone damaged intestinal tissue more seriously. No significant changes was found in the intestinal morphology and function of the mice fed with TMC3115 compared with those in control. However, orally administered TMC3115 also did not enhance the density of ileal villi or the depth of colonic crypts, nor did it improve the proliferation and differentiation of IECs as expected as did the probiotic strains *Lactobacillus paracasei* N1115 and *Lactobacillus rhamnosus* GG in the intestinal development of neonatal mice^29,30^. This might be one of the possible reasons for TMC3115 to not significantly protect mice from the damages by ceftriaxone and vancomycin when combined with them. On the other hand, the spleen index of neonatal mice was significantly increased when vancomycin combined with TMC3115 compared with when administered alone, indicating that TMC3115 might alleviate the systemic immunity of the host by improving the vancomycin damaged spleen index.

In the present study, the fecal microbiota of the neonatal mice under study were detected and profiled with qPCR and NGS of 16sRNA to analyze the changes in intestinal microbiota during treatments with antibiotics and TMC3115. The oral administration of antibiotics and probiotic TMC3115 altered the intestinal microbiota of neonates to a large extent, both quantitatively and qualitatively. However, the microbial shifts caused by ceftriaxone and vancomycin were completely different from each other. Ceftriaxone significantly reduced bacterial counts with an increase in the diversity of fecal microbiota. On the other hand, vancomycin both apparently decreased the diversity and the numbers of fecal microbiota. When TMC3115 was combined with ceftriaxone, it significantly increased bacterial counts with a decrease in the diversity of fecal microbiota compared with ceftriaxone treatment alone. However, no significant changes in the bacterial counts or diversity of fecal microbiota were found in the mice treated with vancomycin alone or combined with TMC3115. Significantly increased fecal microbial diversity with no significant changes in bacterial counts were found in TMC3115 alone treated mice. These results indicate that TMC3115 can alleviate or normalize the antibiotic-induced abnormal intestinal microbiota and this might be one of crucial underlying mechanisms for TMC3115 to mitigate the damages caused by antibiotics to the spleen organ index. Therefore, the supplementation of selected probiotics such as TMC3115 might be practical to reduce the side effects of antibiotics at least partly to intestinal microbiota.

The fecal microbiota of the normal mice in the control group and those fed with TMC3115 were dominated by *Bacteroidetes*. However, *Firmicutes* and *Proteobacteria* dominated the fecal microbiota of the mice treated by ceftriaxone and vancomycin, respectively. These results indicate that each antibiotic can dynamically and specifically alter the fecal microbiota composition, dependent on its spectrum and properties. It is well know that 80% of the identified fecal microbiota can be classified into three dominant phyla: *Bacteroidetes, Firmicutes*, and *Actinobacteria*. In general terms, the *Firmicutes* to *Bacteroidetes* ratio is regarded to be of significant relevance in human gut microbiota composition. Many studies have demonstrated that the proportions of *Firmicutes*:*Bacteroidetes* could be significantly increased in obese people and that the domination of intestine microbiota by *Firmicutes* might be one of the risk factors inducing metabolic disorders in host animals and healthy weight children and adults^40^.

*Proteobacteria* are a small group of gram-negative bacteria mainly composed of lipopolysaccharides and include many potent pathogenic bacteria such as *E. coli*. Recent studies have indicated that an imbalanced gut microbiota often arises from a sustained increase in the abundance of *Proteobacteria* and suggested that an increased prevalence of *Proteobacteria* could be considered a potential diagnostic signature of dysbiosis and risk of disease^41^. Therefore, *Firmicutes*- and *Proteobacteria*-dominated intestinal microbiota caused by ceftriaxone and vancomycin could influence the health of host animals more than the intestinal microbiota themselves.

Differing from ceftriaxone and vancomycin, mice orally fed with TMC3115 did not exhibit significant alterations in the intestinal microbiota. However, TMC3115 revealed a strong ability to modulate the intestinal microbiota of the mice exposed to ceftriaxone by increasing *Actinobacteria* and decreasing *Proteobacteria*. TMC3115 did not influence the intestinal microbiota of the mice exposed to vancomycin, although *Actinobacteria* was slightly increased. One of the possible reasons might be the antibiotic resistance of TMC3115 to ceftriaxone and its sensitivity to vancomycin, which may aid in the colonization of TMC3115 in mice exposed to ceftriaxone but not in those treated with vancomycin. Another key reason may be differences in the modes of antibacterial activity and the spectrum between ceftriaxone and vancomycin. Ceftriaxone may kill most of the normal intestinal flora, resulting in a significant reduction of total bacteria, which can provide a habitat for other potential pathogens like *Staphylococcus* and *Enterococcus*, thereby increasing the diversity of fecal microbiota^42^. Therefore, colonization of the intestine by orally fed TMC3115 in mice may suppress *Proteobacteria*. In light of our findings that ceftriaxone and vancomycin differently and characteristically alter the intestinal bacterial concentration and diversity respectively, TMC3115 could be considered to normalize the intestinal microbiota when they are quantitatively reduced by antibiotics with a relatively broad spectrum, such as ceftriaxone. These results suggest that the benefits of a probiotic may be strain-dependent and that different probiotics should be selected to counteract the side effects of different antibiotics.

Some food stuff that cannot be directly digested and absorbed can be fermented by certain species of intestinal bacteria, especially *Bacteroides*, which degrade complex carbohydrates to their component monosaccharides. The main excreted products of *Bacteroides* are acetate and propionate, two crucial short-chain fatty acids (SCFAs)^43,44^. SCFAs are among the most abundant of the bacterial metabolites, and playing a crucial role as a fuel source for IECs^45^ and exerting effects on intestinal morphology and function^46^. In the present study, neonatal mice treated with ceftriaxone and vancomycin showed severe intestinal tissue damage, IECs dysfunction, significantly decreased production of SCFAs and greatly depleted *Bacteroides* populations. Moreover, TMC3115 were found to significantly increase the production of SCFAs. The underlying mechanism might be the significantly decreased/or increased *Bacteroides* which, in turn, reduces/or promotes the amount of SCFAs produced. These results indicate once again that dysbiosis of intestinal microbiota in early life might negatively influence the development of intestinal tissue and its functional differentiation, and oral administration of probiotics could improve the intestinal microbiota composition through promoting the production of SCFAs although no obvious improved morphological changes were observed.

After termination of treatment with antibiotics or the probiotic, the neonatal mice grew well. There was no significant difference in body weight, systemic or local inflammation, or other clinical symptoms among the adult mice. The fecal bacterial counts become almost the same as each other at the adult stage. However, the diversity of the fecal microbiota in the mice exposed to antibiotics was apparently lower than those mice not exposed to antibiotics. Furthermore, the fecal microbiota of the antibiotic-treated mice remained characterized with less *Bacteroidetes* and more *Firmicutes* compared with the control mice at the adult stage, although a vast increase was found. Furthermore, there were significant differences in the predominant bacterial composition of fecal microbiota at the genus level. These results indicate that the impaired intestinal microbiota of host animals in early life might not be recovered or repaired even in the adult stage, although no morphological or cell function damage was observed in the intestinal tract of adult mice as observed in the neonatal stages. Therefore, we can conclude that intestinal tissue damage caused by antibiotic treatment in early life could be restored after the termination of antibiotic exposure. Additionally, the production of the seven types of SCFAs increased similar to control group in adult stage. These results could suggest that *Bacteroides* becoming predominance in intestinal microbiota of the adult might be the underlying mechanism for the increase of production of SCFAs, because these bacteria can shapes a virtuous cycle for SCFAs production thus increasing the amount of fuel available for IECs. However, the abnormal intestinal microbiota caused by antibiotics in early life cannot be restored to the same composition as those of normal intestinal microbiota. Thus, damages to the intestinal microbiota of neonates by antibiotics could negatively influence the health of the host animal in the neonatal stage. Furthermore, this could have lasting consequences on the health of host animals, even in adults, because the impaired intestinal microbiota from the neonatal stage might remain in adults or change the intestinal microbiota composition of adults in an abnormal manner.

IgE has been considered a biomarker of many allergic diseases, and IgE production is dependent on CD4+ T cells^13^. In the present study, at PND21, the terminated point of antibiotics treatment, before antigen (OVA) stimulation, orally fed ceftriaxone and vancomycin significantly decreased the serum total IgE levels of the tested mice. Moreover, the mice treated with antibiotics combined TMC3115 and TMC3115 alone showed similar serum total IgE level to control. These results suggested that early life antibiotics treatment could influence postnatal immune development characterized with immature immune function. These was different from the findings in the studies with germ free mice^13^. The germ free mice expressed higher IgE level than did conventional mice under normal condition without antigen attack^11^. In the present study, the altered specific microbiota induced by antibiotics might induce an intermediate state of the tested mice between totally germ free and relatively normal microbiota, resulting in such a different immunity with serum total IgE level related to immune cells unbalance^22^, TMC3115 showed protective effects both on microbiota community and immune development during early life.

Differing from the weaning stage, after antigen stimulation, neonatal ceftriaxone treatment, but not vancomycin significantly enhanced the serum total IgE and OVA-specific IgE production of mice late in adult. The antigen stimulation may be the key trigger in the dynamic changes of serum total IgE level in antibiotics treated mice from weaning to adulthood. The intervention of TMC3115 to neonates significantly reduced the serum total IgE of them late in adult. However, such protective effect of TMC3115 to IgE production was not observed when combined TMC3115 with ceftriaxone. These results indicate that exposure of some kinds of antibiotics such as ceftriaxone in early life can enhanced IgE mediated allergic response of the host animal characterized with increased serum IgE production. These results also indicate that bifidobacteria, especially some of selective strains like TMC3115 used in the neonatal stage, might protect host animal from IgE hypersensitivity and reduce the risk of IgE medicated allergic disorders. However, such benefits of bifidobacteria might be obliterated by antibiotic such as ceftriaxone.

In order to explore the underlying mechanism related to antibiotic such as ceftriaxone to influence the IgE mediated allergic disorders in adulthood, the splenic lymphocytes, serum cytokines were also profiled in the present study. CD4+ T cells recognize peptides presented on major histocompatibility complex class II molecules found on antigen presenting cells (APCs), and they play a major role in instigating and shaping adaptive immune responses. CD4+ T cells mainly consist of T-helper (Th) cells Th1, Th2, and Th17, and regulatory T-cells (Treg), which could be induced by different commensal microbiota. For example, *Listeria* drives a Th1 response, *Nematodes* drive Th2 responses, *segmented filamentous bacteria* drive Th17 responses, and *altered Schaedler flora*, as well as consortia of *Clostridia* drive Treg responses^46^. Furthermore, CD4+ T cells subsets were found to be altered in spleen and gut-associated lymphoid tissues with ceftriaxone or vancomycin treatment^12,47^. One germ-free mice study found that microbiota diversity is an essential factor preventing the induction of hyper-IgE syndrome^13^. In this study, mice treated with ceftriaxone early in life showed higher levels of IgE and CD4+ T cells than control and TMC3115 group. These results demonstrate that neonatal ceftriaxone treatment can enhance serum IgE levels and alter CD4+ T cell differentiation in the adult stage and the probiotic TMC3115 may alleviative these effects. The underlying mechanism might be the lack of a specific intestinal bacteria to induce CD4+ T cell subsets response, which may be related to the reduced microbiota diversity and damaged composition caused by early life ceftriaxone treatment. Probiotic TMC3115 is, therefore, a potential strain to alleviate IgE-mediated allergic disorders via intestinal microbiota modulation, which might be especially critical in early life.

In the present study, the apparent changes were observed among the tested mice of adulthood in the microbial populations, serum total IgE/or antigen specific IgE levels, and CD4+ T cells differentiation of the host animal. These results suggested the overall impact of different kinds of early life antibiotics/or probiotic on host microbial community networks and the conjunction with host immunity, even in adulthood. However, further studies are still necessary to determine how intestinal microbiota of early life could affect the immunity of host animal in adulthood.

In obese human subjects, less *Bacteroidetes* and more Firmicutes were characteristic of the intestinal microbiota^48^. In a recent study with Japanese cedar pollinosis patients, *Bacteroidetes* showed positive correlation with LDL- and HDL-cholesterol levels, whereas *Firmicutes* showed negative correlation with total cholesterol, LDL- and HDL- cholesterol, indicating the possible correlation between allergic disease and lipid metabolisms^49^. Interestingly, in this study, mice with a higher IgE level had fewer *Bacteroidetes* and more *Firmicutes* in their intestinal microbiota than the control. It could, therefore, be possible that infants with a high IgE level may develop obesity due to the distinctive microbiota community. Further studies focusing on this point may be of critical significance.

In conclusion, antibiotics and TMC3115 can modulate intestinal microbiota and the production of SCFAs with different manners, affecting the development and function of the intestinal epithelium and immunity to different degrees in neonatal mice. Neonatal ceftriaxone-induced abnormal intestinal microbiota, epithelium and immunity could last to adulthood partly, which might be associated with the enhancement of host susceptibility to IgE-mediated allergies and related immune responses, TMC3115 may protect against the side effects of antibiotic treatment, at least partly.

## Methods

### Mice

Twenty-four pregnant specific pathogen-free BALB/c mice at day 13 of gestation were purchased from the Institute of Laboratory Animals at the Sichuan Academy of Medical Sciences & Sichuan Provincial People’s Hospital (Sichuan, PR China) and kept in individually ventilated plastic cages at an ambient temperature of 23 ± 1 °C and humidity of 50–70% under a 12 h light/dark cycle with free access to water and food. Immediately after delivery, litters of 24 dams were randomly assigned and segregated by treatment group, with 18 mice per treatment group. All experimental procedures were performed in accordance with the Guidelines for Animal Experiments at West China School of Public Health, Sichuan University (Sichuan, PR China). The animal experiment facility and animals used for the present study were officially approved by the Experimental Animal Management Committee of Sichuan Government (Approved number: SYXK2013-011). The experimental protocols were approved by the West China School of Public Health Medical Ethics Committee of Sichuan University (Sichuan, PR China).

### Antibiotic and Probiotic treatment

Ceftriaxone and vancomycin (Aladdin Shanghai Biochemical Technology, Shanghai,.PR China) were dissolved in saline at 100 mg/kg. The reasons for choosing these two antibiotics were as previously described^22^.

TMC3115 was kindly supplied by Hebei Inatural Biotech Co. Ltd. (Hebei, PR China). The freeze-dried living TMC3115 (3.4 × 10^12^ colony-forming unit [CFU]/g) was dissolved in sterile saline to prepare bacterial suspensions of 10^11^ CFU/ml, 10^10^ CFU/ml, and 5 × 10^9^ CFU/ml, respectively. The daily intake of living TMC3115 was estimated as 10^9^ CFU/mouse.

One hundred and eight neonatal mice were orally gavaged with antibiotics or probiotic daily by 24-gauge feeding needles attached to a 1-ml syringe (Instech Laboratories, Inc., PA, USA) at 10 µL of the dilution from postnatal day (PND) 0–7, 100 µL from PND 7–14, and 200 µL from PND 14–21. Gavage was discontinued after PND 21 (Fig. 1a).

### OVA immune sensitization

OVA sensitization was induced with methods previously described^50^ with minor modifications. Briefly, mice were sensitized intraperitoneally with 40 µg OVA and 4 mg Imject Alum (Sigma-Aldrich, MO, USA) on PND 22, 36, 43, and 50 (Fig. 1a).

### Detection of total serum IgE, OVA-specific IgE and serum cytokines

Mice were sacrificed at PND 21 and PND 56, and blood samples were collected. The sera were isolated and frozen at −80 °C as described previously^22^. Total serum IgE was measured by enzyme-linked immunosorbent assay (ELISA) (Invitrogen, Thermo Fisher Scientific, Inc., MA, USA). Serum OVA-specific IgE level was detected by ELISA (DS Pharma Biomedical Co., Ltd., Osaka, Japan). Serum TNF-α, IL-2, IL-4, IL-6, IL-10, IL-12, IL-17A and IFN-γ levels were determined by a Luminex assay (R&D Systems Inc., MN, USA). Assays were performed according to the manufacturer’s instructions and read using a Multiskan™ GO microplate spectrophotometer (Thermo Fisher Scientific, Inc., MA, USA) and a Luminex 200^TM^ multiplexing instrument (Merck Millipore, MA, USA).

### Histopathology

Whole intestines collected from mice at PND 21 and 56 were fixed in 10% neutral phosphate-buffered saline formalin for 24 h and routinely stained with hematoxylin and eosin (H&E). Optical microscopic images were inspected by a pathologist blinded to the experimental design. Thereafter, the depths of at least 20 villi or crypts in the ileum and colon were measured for each mouse with no less than 12 fields the same as that used in a previous study^22^.

### Immunohistochemistry

Paraffin-embedded slides were cut into 5-μm widths, deparaffinized in xylene, and rehydrated with graded ethanol as described previously^22^. Antigen retrieval was performed by heating the tissue slides to a high temperature in antigen retrieval buffer (ethylenediaminetetra acetic acid for Ki67 staining, pH 9.0; citrate acid for Muc2 staining, pH 6.0) followed by incubation with 3% H_2_O_2_ (Servicebio Technology Co. Ltd., Wuhan, PR China) for 25 min at room temperature while protected from light. Then, the slides were blocked with 3% bovine albumin for 30 min at room temperature. Thereafter, the slides were incubated overnight with primary antibodies at 4 °C. The primary antibodies applied for staining and their respective dilutions were as follows: rabbit-polyclonal anti-mouse Ki67 antibody (1:500 dilution; Servicebio Technology Co. Ltd.), and rabbit-polyclonal anti-mouse Muc2 (1:500 dilution; Abcam, MA, USA). The slides were then permeated with a horseradish peroxidase-conjugated secondary antibody (1:200 dilution; Servicebio Technology Co. Ltd.) for 50 min at room temperature, washed with phosphate-buffered saline three times, stained with the DAB^+^ Substrate Chromogen System (Servicebio Technology Co. Ltd.) for 1–5 min, and then counterstained with hematoxylin. Slides were scanned using the Pannoramic MIDI II automatic digital slide scanner (3DHISTECH Ltd., Budapest, Hungary) and five 400-fold magnified images from each slide were selected from left to right by CaseViewer 2.0.2.61392 (3DHISTECH Ltd.). Image-Pro Plus 6.0 software (Media Cybernetics, Inc., MD, USA) was used to select the identical brownish yellow as a uniform standard for determining the positive response, and the integral optical density of positive responses in each image was measured.

### Flow cytometry

Spleen tissues of mice at PND 56 were collected. Cell isolation and CD4+ T cell subsets staining were performed as previously described^22^.

### DNA extraction and bacterial quantitation

Fresh Stool pellets of mice at PND 21 and 56 were collected and frozen at −80 °C. Total DNA was extracted using the TIANamp Stool DNA Kit (Tiangen Biotech Co. Ltd., Beijing, PR China) in strict accordance with the manufacturer’s instructions. Quantitative polymerase chain reaction (qPCR) was performed on the isolated bacteria as previously described^22^. And the primers are the 16S rRNA V6-V8 universal bacterial primers: U968-F- 5′-AAC GCG AAG AAC CTT AC-3′ and L1401-R-5′-CGG TGT GTA CAA GAC CC-3′. 16S rDNA copies were calculated as total bacterial counts.

### Amplification and sequencing of genes encoding 16S rRNA

The 5′ ends of the primers were tagged with unique sample-specific identifiers (barcodes) and sequenced with universal primers^51^ (forward primer: V3-338F 5′-ACTCCTACGGGAGGCAGCAG-3′ and reverse primer V4-806R 5′-GGACTACHVGGGTWTCTAAT-3′). PCR amplification was performed in a total volume of 25 μL reaction mixture containing 50 ng of template DNA, 12.5 μL Phusion® Hot Start Flex 2X Master Mix (New England Biolabs Inc., MA, USA), and 2.5 μL each of primer. Ultra-pure water was used to adjust the final volume. The PCR cycling conditions were as follows: initial denaturation at 98 °C for 30 s, followed by 35 cycles of denaturation at 98 °C for 10 s, annealing at 54 °C for 30 s, and extension at 72 °C for 45 s. Final extension at 72 °C was for 10 min. The PCR products were confirmed by 2% agarose gel electrophoresis and purified using the Agencourt® AMPure® XP System (Beckman Coulter Genomics, MA, USA). The size and quantity of the amplicon library were assessed and sequenced on an Illumina MiSeq instrument (Illumina Inc., CA, USA).

### Bioinformatics

Briefly, the amplicon read processing pipeline QIIME 1.9.1 was used^52^. After filtering the sequences and removing the potential chimeras, high-quality reads were clustered into operational taxonomic units (OTUs) at 97% similarity using the *de novo* UCLUST algorithm^53^. Taxonomic assignment was performed with Greengenes database 13.8^54^, and the OTU abundance table was constructed with QIIME python scripts. Multiple sequence alignment was conducted using the PyNAST 1.2.2 software^55^, and phylogenetic trees were constructed in FastTree 2.1.90 to study the phylogenetic relationships of different OTUs^56^.

Bacterial OTUs with overall relative abundance <0.001% were excluded for downstream analyses. Relative abundance of a microbe in a sample was calculated as the read count normalized by the total reads in that sample, and the microbes with a relative abundance <1% in all samples were classified as “others.” The phylogenetic tree and modified relative abundance tables generated were used to calculate microbial alpha diversity (phylogenetic diversity) with QIIME script and beta-diversity (unweighted and weighted UniFrac distance) in Phyloseq. 1.20.0^57^. Both the microbial composition of groups and the principal coordinates analysis (PCoA) were visualized using R 3.4.1.

Variations of microbes in abundance at different taxonomic ranks were determined using Metastats^56,58^. Metastats was performed using EDDA R-package 1.10.0 and a probability (*P*) value < 0.05 was considered statistically significant^59^.

### Analysis of cecal SCFAs

The analysis of cecal SCFAs was conducted using gas chromatography–mass spectrometry. Supernatants from 1.25 ml of the homogenized cecal samples were obtained by centrifugation (17949 g, 10 min). A gas chromatograph-mass spectrometer Agilent 7890 A/5975 C (Agilent Technologies, Inc.USA) was used for quantification. 4-Methyl valeric acid was used as an internal standard.

### Statistical analysis

All statistical analyses were performed using SPSS 19.0 software (SPSS, Inc., IL, USA). The data are expressed as the mean ± standard error of the mean. One-way ANOVA or the Kruskal–Wallis *H*-test was used for multiple comparisons, and *post hoc* tests using the least significant difference analysis were performed for pairwise comparisons. A probability (*P*) value < 0.05 was considered statistically significant. All statistical tests were two-tailed.

## Electronic supplementary material

Supplementary information

## Data Availability

16S sequencing rawdata for this study have been deposited at Sequence Read Archive (SRA) database in NCBI under the accession number SRP151093 (https://www.ncbi.nlm.nih.gov/sra/SRP151093). Other datasets generated and analyzed during the present study are available from the corresponding author on reasonable request.
