# Supplementary material for: Loading ceftriaxone, vancomycin, and Bifidobacteria bifidum TMC3115 to neonatal mice could differently and consequently affect intestinal microbiota and immunity in adulthood
Source: Sci Rep. 2019 Mar 1;9:3254. doi: 10.1038/s41598-018-35737-1 (PMC6397183; doi:10.1038/s41598-018-35737-1)
Supplement: Supplementary file 1 — Supplementary information [file 41598_2018_35737_MOESM1_ESM.pdf]

Supplementary information

Loading ceftriaxone, vancomycin, and *Bifidobacteria bifidum* TMC3115 to neonatal mice could differently and consequently affect intestinal microbiota and immunity in adulthood

RuYue Cheng[1], JiaWen Guo[1], FangFang Pu[1], ChaoMin Wan[2], Lei Shi[3], Huawen Li[4], YuHong Yang[4], ChengYu Huang[1], Ming Li[1], Fang He[*1]**

[1]Department of Nutrition, Food Hygiene and Toxicology, West China School of Public Health and Healthy Food Evaluation Research Center, Sichuan University, 610041 Chengdu, Sichuan, PR China

[2]Department of Pediatrics of Western China Second Hospital of Sichuan University, Key Laboratory of Birth Defects and Related Diseases of Women a nd Children, 610041, Chengdu, Sichuan, PR China

[3]Department of Clinical Nutrition, West China Hospital, Sichuan University, 610041 Chengdu, Sichuan, PR China

[4] Hebei Inatural Biotech Co., Ltd. 050000 Shijiazhuang, Hebei, PR China.

*Correspondence:

Fang He

E-mail: hf18602880124@163.com

**Co- Correspondence:

Ming Li

E-mail: ldmancheng@163.com

Funding information: National Natural Science Foundation of China (Grant number 81372982).

Table S1 Growth rate of mice at PND 28 and PND35

Group	PND28/21	PND35/21
Control	1.22093±0.049172697 a	1.754654±0.056296959 a
Ceftriaxone	1.227212±0.028876946 a	1.550802±0.051420144 bc
Vancomycin	1.281697±0.024769572 a	1.737158±0.07036476 ab
TMC3115	1.300216±0.052911482 a	1.688636±0.096658585 ab
Ceftri+TMC3115	0.995577±0.02582756 b	1.436436±0.057417893 c
Vanco+TMC3115	1.068182±0.026875634 b	1.492862±0.05819838 c

Fold changes of murine body weight at PND28 and 35 verse PND21 were calculated and taken as growth rate (n=12/group). Data are expressed as mean ± standard error of the mean. Mean values not sharing a letter are significantly different. N, number; TMC3115, *Bifidobacterium bifidum* TMC3115; Ceftri, Ceftriaxone; Vanco, Vancomycin. PND, postnatal day.

Table S2 Top 10 predominant bacteria in different groups at genus level PND21

Rank	Control	Ceftriaxone	Vancomycin	TMC3115	Ceftri+TMC3115	Vanco+TMC3115
1	*Bacteroides*	*Staphylococcus*	*Lactobacillus*	*Bacteroides*	*Enterococcus*	*Lactobacillus*
	55.29%	35.14%	94.36%	57.78%	78.04%	96.29%
2	*Lactobacillus*	*Enterococcus*	*Enterobacter*	*[Prevotella]*	*Bifidobacterium*	*Enterobacter*
	31.8%	18.91%	3.64%	16.34%	12.74%	2.07%
3	*Parabacteroides*	*Rummeliibacillus*	*Pseudomonas*	*Parabacteroides*	*Rummeliibacillus*	*Bifidobacterium*
	6.46%	16.55%	0.82%	11.32%	3.14%	0.42%
4	*[Prevotella]*	*Acinetobacter*	*Klebsiella*	*Lactobacillus*	*Staphylococcus*	*Others*
	1.99%	9.45%	0.48%	5.86%	1.68%	0.24%
5	*Anaeroplasma*	*Stenotrophomonas*	*Others*	*Odoribacter*	*Others*	*Acinetobacter*
	1.5%	5.91%	0.28%	2.85%	1.21%	0.2%
6	*Stenotrophomonas*	*Others*	*Bacteroides*	*Oscillospira*	*Stenotrophomonas*	*Stenotrophomonas*
	1.28%	3.88%	0.14%	1.21%	1.01%	0.18%
7	*Enterococcus*	*Vibrio*	*Enterococcus*	*Prevotella*	*Vagococcus*	*Klebsiella*
	0.95%	2.51%	0.13%	1.13%	0.74%	0.16%
8	*Others*	*Arthrobacter*	*Bifidobacterium*	*Helicobacter*	*Wautersiella*	*Pseudomonas*
	0.25%	1.64%	0.03%	0.96%	0.54%	0.1%
9	*Oscillospira*	*Lactobacillus*	*Parabacteroides*	*AF12*	*Acinetobacter*	*Bacteroides*
	0.11%	1.22%	0.03%	0.7%	0.44%	0.08%
10	*Prevotella*	*Bacteroides*	*[Prevotella]*	*Others*	*Bacteroides/Lactobacillus*	*Wautersiella*

	0.11%	1.21%	0.03%	0.49%	0.12%	0.07%

The percentage is the mean relative abundance of different genera. n=4/group. TMC3115*, Bifidobacterium bifidum* TMC3115; Ceftri, Ceftriaxone; Vanco, Vancomycin; PND, postnatal day.

Table S3 Top 10 predominant bacteria in different groups at genus level PND56

Rank	Control	Ceftriaxone	Vancomycin	TMC3115	Ceftri+TMC3115	Vanco+TMC3115
1	*Bacteroides*	*Bacteroides*	*Oscillospira*	*Bacteroides*	*Bacteroides*	*Bacteroides*
	36.3%	38.41%	23.91%	31.73%	66.4%	61.28%
2	*[Prevotella]*	*Oscillospira*	*Lactobacillus*	*Oscillospira*	*Parabacteroides*	*Parabacteroides*
	13.72%	18.98%	23.27%	21.67%	13.2%	22.65%
3	*Lactobacillus*	*Parabacteroides*	*Mucispirillum*	*Lactobacillus*	*Akkermansia*	*Akkermansia*
	12.86%	14.71%	9.86%	9.55%	5.46%	7.78%
4	*Parabacteroides*	*Akkermansia*	*Ruminococcus*	*[Prevotella]*	*[Prevotella]*	*Trabulsiella*
	11.97%	11.33%	7.93%	7.32%	4.57%	1.8%
5	*Prevotella*	*[Prevotella]*	*Bacteroides*	*Ruminococcus*	*Lactobacillus*	*Oscillospira*
	5.38%	6.63%	6.97%	6.14%	3.2%	1.7%
6	*Oscillospira*	*Desulfovibrio*	*Helicobacter*	*Parabacteroides*	*Sutterella*	*Blautia*
	4.91%	2.5%	6.36%	3.09%	1.6%	1.55%
7	*Helicobacter*	*Bilophila*	*[Prevotella]*	*Bilophila*	*Oscillospira*	*Klebsiella*
	2.06%	2.26%	5.77%	2.61%	1.36%	0.16%
8	*Odoribacter*	*Ruminococcus*	*Bilophila*	*Odoribacter*	*Helicobacter*	*Bilophila*

	1.95%	1.55%	2.87%	2.05%	1.13%	0.82%
9	*Sutterella*	*Dehalobacterium*	*Adlercreutzia*	*Coprococcus*	*Trabulsiella*	*Enterobacter*
	1.4%	0.79%	1.88%	1.93%	1.02%	0.64%
10	*Akkermansia*	*Coprococcus*	*[Ruminococcus]*	*Dehalobacterium*	*Blautia*	*Helicobacter*
	1.34%	0.71%	1.69%	1.88%	0.65%	0.59%

The percentage is the mean relative abundance of different genera. n=4/group. TMC3115*, Bifidobacterium bifidum* TMC3115; Ceftri, Ceftriaxone; Vanco, Vancomycin; PND, postnatal day.

Table S4 Short chain fatty acids in cecal contents of mice at PND 56

SCFAs (μg/g)	Control	Ceftriaxone	Vancomycin	TMC3115	Ceftri+TMC3115	Vanco+TMC3115
Acetic acid	1167.38±465.87	1217.62±410.50	1057.22±127.36	1284.02±281.19	1677.28±875.18	1124.22±260.79
Propionic acid	495.46±170.90	412.92±124.93	405.22±145.91	497.97±144.25	657.53±313.52	657.53±313.52
Isobutyric acid	68.50±31.34	65.60±18.55	69.77±13.55	71.89±17.17	60.53±16.70	41.65±5.63
Butyric acid	858.63±552.98	1075.77±895.05	858.63±552.98	665.60±181.71	325.51±130.00	188.55±46.06
Isovaleric acid	51.49±26.04	49.54±21.04	54.80±15.85	51.93±14.21	38.94±12.66	38.24±8.14
Valeric acid	134.85±37.53	85.53±89.12	106.26±26.83	87.44±18.03	2.5±0.42	ND
Caproic acid	2.92±1.27	2.10±1.51	2.21±0.51	2.39±1.64	0.62±0.68	0.21±0.10

n=4, per group. Data are expressed as mean ± standard deviation (SD) of mean. SCFAs: short chain fatty acids; TMC3115*, Bifidobacterium bifidum* TMC3115; Ceftri, Ceftriaxone; Vanco, Vancomycin ; PND, postnatal day, μg: microgram; ND, not detected.

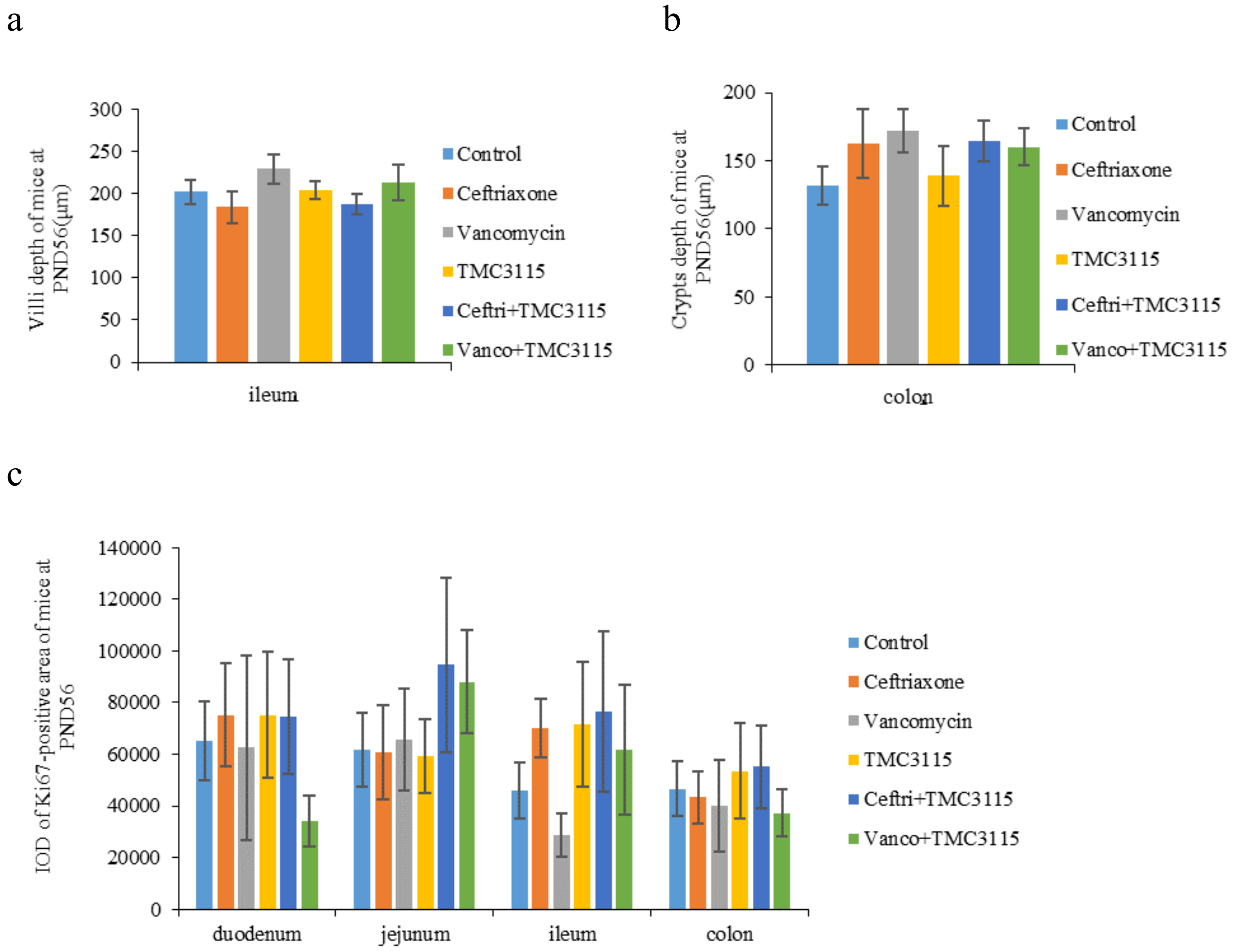
a
300
250
200
150
100
50
0
Villi depth of mice at PND56(μm)
ileum
Control
Ceftriaxone
Vancomycin
TMC3115
Ceftri+TMC3115
Vanco+TMC3115
b
200
150
100
50
0
Crypts depth of mice at PND56(μm)
colon
Control
Ceftriaxone
Vancomycin
TMC3115
Ceftri+TMC3115
Vanco+TMC3115
c
140000
120000
100000
80000
60000
40000
20000
0
IOD of Ki67-positive area of mice at PND56
duodenum
jejunum
ileum
colon
Control
Ceftriaxone
Vancomycin
TMC3115
Ceftri+TMC3115
Vanco+TMC3115

d

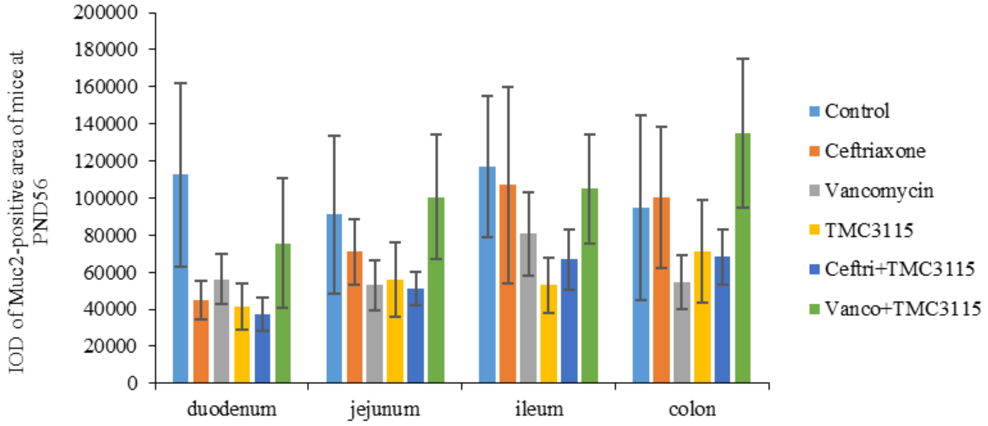

IOD of Muc2-positive area of mice at PND56
200000
180000
160000
140000
120000
100000
80000
60000
40000
20000
0
duodenum
jejunum
ileum
colon
Control
Ceftriaxone
Vancomycin
TMC3115
Ceftri+TMC3115
Vanco+TMC3115

e

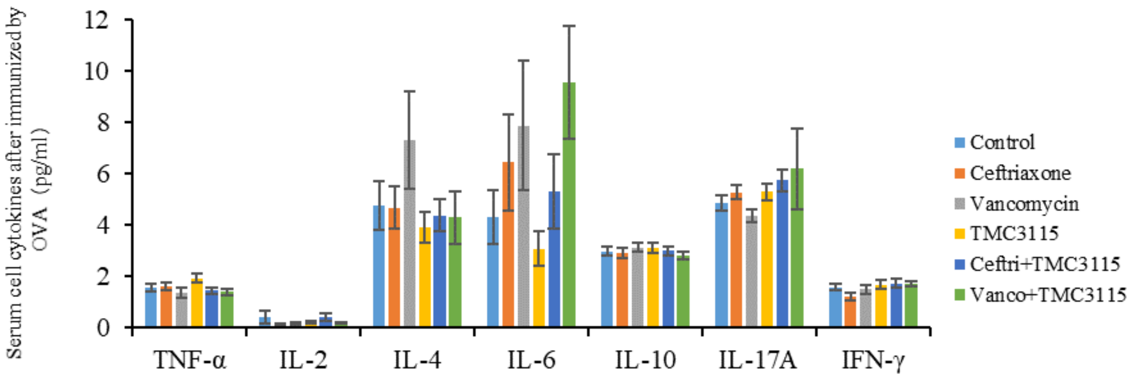

Serum cell cytokines after immunized by OVA (pg/ml)
12
10
8
6
4
2
0
TNF-α
IL-2
IL-4
IL-6
IL-10
IL-17A
IFN-γ
Control
Ceftriaxone
Vancomycin
TMC3115
Ceftri+TMC3115
Vanco+TMC3115

Fig.S1 Intestinal tissue injury of mice at PND56. (a) The depth of intestinal villi in ileum of mice at PND56 (n=12/group). (b) The depth of crypts in colon of mice at PND56 (n=12/group). (c) Ki67-positive area of the four intestinal segments (duodenum, jejunum, ileum, and colon) in mice at PND56. (d) Muc2-positive area of the four intestinal segments (duodenum, jejunum, ileum, and colon) in mice at PND56 (n=12/group). There was no significant difference between groups of the ileal villi depth, colonic crypts depth and the Ki67/Muc2-positive area of the four segments. IOD: integral optical density. (e) Serum cell cytokines of mice at PND56 (n=12/group). TMC3115*, Bifidobacterium bifidum* TMC3115; Ceftri, Ceftriaxone; Vanco, Vancomycin. PND, postnatal day.
